# Exercise-induced Nogo-A influences rodent motor learning in a time-dependent manner

**DOI:** 10.1371/journal.pone.0250743

**Published:** 2021-05-05

**Authors:** Jörg H. Stehle, Zhiyuan Sheng, Laura Hausmann, Philipp Bechstein, Oliver Weinmann, Juha Hernesniemi, Joseph S. Neimat, Martin E. Schwab, Ajmal Zemmar

**Affiliations:** 1 Department of Neurosurgery, Henan Provincial People´s Hospital, Henan University People’s Hospital, Henan University School of Medicine, People’s Hospital of Zhengzhou University, Zhengzhou, China; 2 Dr. Senckenbergische Anatomie, Goethe-University Frankfurt, Frankfurt am Main, Germany; 3 Department of Neurology, University Hospital RWTH Aachen, Aachen, Germany; 4 Brain Research Institute, University of Zurich, Zurich, Switzerland; 5 Department of Biology and Department of Health Sciences and Technology, ETH Zurich, Zurich, Switzerland; 6 Department of Neurosurgery, University of Louisville, School of Medicine, Louisville, Kentucky, United States of America; Bilkent University, TURKEY

## Abstract

The adult, mature central nervous system (CNS) has limited plasticity. Physical exercising can counteract this limitation by inducing plasticity and fostering processes such as learning, memory consolidation and formation. Little is known about the molecular factors that govern these mechanisms, and how they are connected with exercise. In this study, we used immunohistochemical and behavioral analyses to investigate how running wheel exercise affects expression of the neuronal plasticity-inhibiting protein Nogo-A in the rat cortex, and how it influences motor learning *in vivo*. Following one week of exercise, rats exhibited a decrease in Nogo-A levels, selectively in motor cortex layer 2/3, but not in layer 5. Nogo-A protein levels returned to baseline after two weeks of running wheel exercise. In a skilled motor task (forelimb-reaching), administration of Nogo-A function-blocking antibodies over the course of the first training week led to improved motor learning. By contrast, Nogo-A antibody application over two weeks of training resulted in impaired learning. Our findings imply a bimodal, time-dependent function of Nogo-A in exercise-induced neuronal plasticity: While an activity-induced suppression of the plasticity-inhibiting protein Nogo-A appears initially beneficial for enhanced motor learning, presumably by allowing greater plasticity in establishing novel synaptic connections, this process is not sustained throughout continued exercise. Instead, upregulation of Nogo-A over the course of the second week of running wheel exercise in rats implies that Nogo-A is required for consolidation of acquired motor skills during the delayed memory consolidation process, possibly by inhibiting ongoing neuronal morphological reorganization to stabilize established synaptic pathways. Our findings suggest that Nogo-A downregulation allows leaning to occur, i.e. opens a ‘learning window’, while its later upregulation stabilizes the learnt engrams. These findings underline the importance of appropriately timing of application of Nogo-A antibodies in future clinical trials that aim to foster memory performance while avoiding adverse effects.

## Introduction

While the central nervous system (CNS) exhibits a high degree of plasticity during early development, it becomes increasingly hard-wired and more stable with age. Exercise can counteract this limitation, and maintain or even induce plasticity in the adult CNS, e.g. by increasing the number of new neurons [1–3], by neovascularisation [4], by enhancing synaptic plasticity and plasticity-related genes [5, 6], by increasing dendritic complexity and spine density [7], improving cognitive function [8], learning and memory [9] and functional recovery [10]. Beyond the observational level, little is known about molecular factors either stimulating or limiting exercise-induced neuronal plasticity. One such candidate factor is Nogo-A, originally identified as a myelin-derived inhibitor of axonal outgrowth, which was originally shown to be involved in axonal regeneration and enhanced functional recovery after spinal cord injury [11–13]. Today it is known that Nogo-A is not only expressed in oligodendroglia but also in neurons [14–18]. Blockade of Nogo-A function with antibodies results in enhanced axonal and dendritic outgrowth and improves functional recovery after spinal cord injury and stroke [13, 19, 20]. Nogo-A also acts as a repressor of functional and structural plasticity in the non-injured central nervous system, influencing neurogenesis [21], angiogenesis [22], the expression of plasticity-related genes [23, 24] and modulation of synaptic plasticity [25]. Effects of Nogo-A on brain plasticity have also been shown in the murine motor cortex [26]: While on the one hand, downregulation of Nogo-A enhanced acquisition of novel motor tasks [14, 26], on the other hand upregulation of Nogo-A signaling is required to consolidate memories [27–29].

In this study, we studied the regulation of Nogo-A during exercise-induced motor learning. We found Nogo-A expression is regulated in a stringently time-dependent, biphasic manner: it is downregulated in the motor cortex immediately following exercise to promote CNS plasticity, as described [29], and is upregulated again at later stages to serve its known function as a memory consolidator [27–29]. This understanding opens new avenues to strategies targeting Nogo-A-mediated plasticity for neuro-regeneration after CNS injury, and highlights the relevance of optimal timing when applying such antibodies in clinical approaches.

## Materials and methods

### Animals

Male adult Sprague-Dawley rats (Janvier, France, RGD catalogue number 38676310, RRID: RGD_38676310; strain: RjHan:SD; www.janvier-labs.com/en/fiche_produit/sprague_dawley_rat/) of 180–220 g weight were used for all experiments. All procedures described in the present study were approved by the Cantonal Veterinary Office in Zurich and carried out in accordance with general principles of Laboratory Animal Care such as handling animals with caution and care, or applying anesthesia prior to painful procedures.

**Fig 1:**
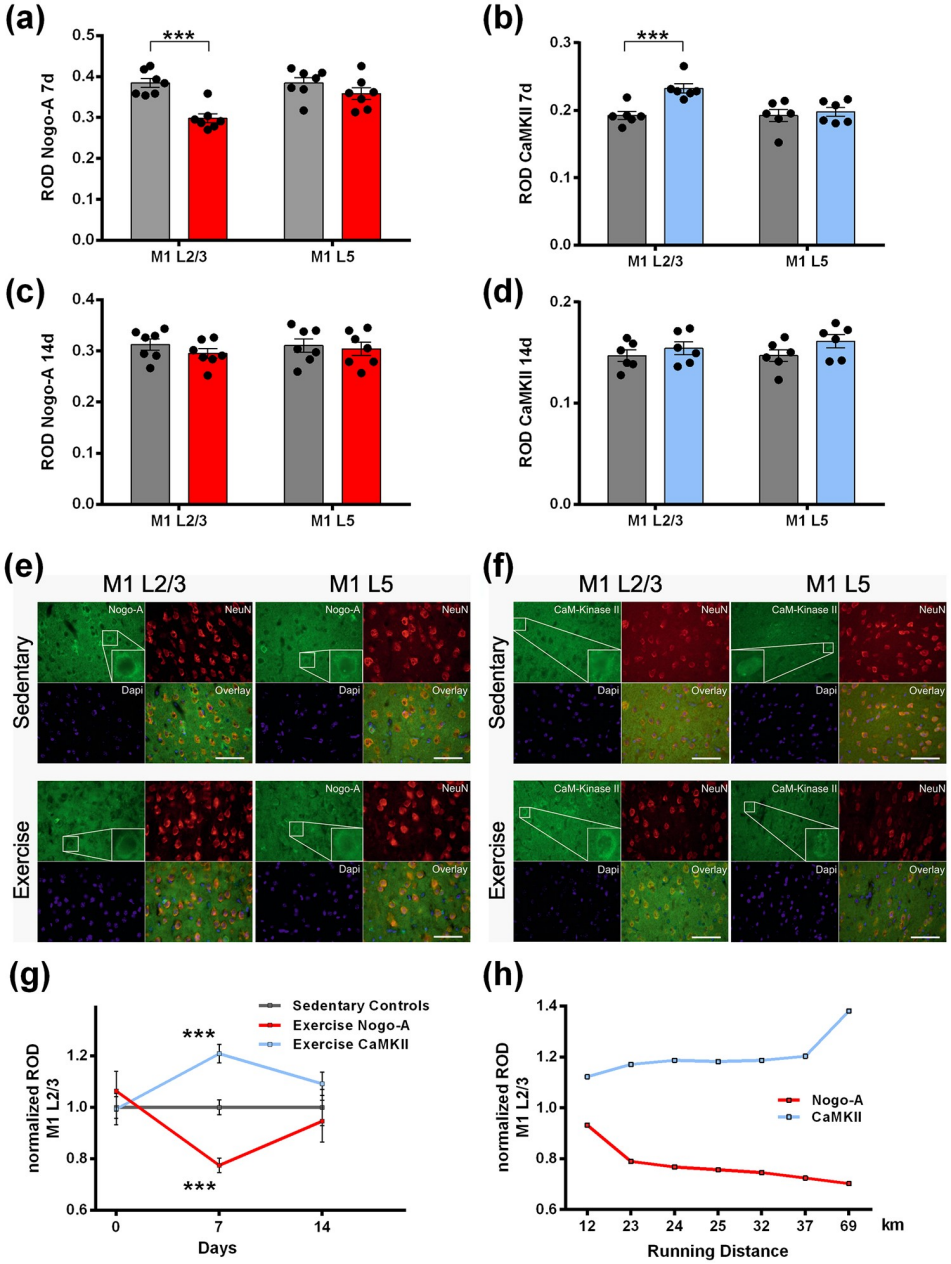
Modulation of Nogo-A and CaM Kinase II protein levels by running wheel exercise in primary motor cortex. **(a-d)** Semiquantitative densitometric analyses of immunohistochemical staining. Sedentary control rats (gray columns) were compared with running wheel exercised animals (Nogo-A: red columns; CamKII: blue columns) and shown as relative optical density (ROD) ± SEM. Nogo-A (n = 7) and CaM Kinase II (CamKII; n = 6) were assessed after 7 days (a,b) and 14 days (c,d), both in M1 layers 2/3 (M1 L2/3) and 5 (M1 L5), respectively. After 7 days of exercise, Nogo-A protein levels significantly decreased in M1 L2/3 (***: *P* = 0.001), but not in M1 L5 (a). Conversely, after 7 days of exercise CamKII levels significantly increased in M1 L2/3 (***: *P* = 0.002) but not in M1 L5 (b). After 14 days of running wheel exercise (c,d) no changes were observed compared to control for both, Nogo-A (n = 7) or CamKII (n = 6), despite slightly overall lower ROD values. Individual values are indicated by black dots. **(e-f)** Representative images for immunostaining against Nogo-A (e) and CamKII (f) for data shown in (a,b). In each subsection, additional stainings are shown for neuronal marker NeuN (red), the nuclear marker Dapi (blue) and an overlay image of both signals in M1 L2/3 and M1 L5, respectively. For better visibility an exemplary Nogo-A positive neuron is magnified. Scale bar: 60 μm. **(g)** Temporal representation of data shown in (a-f). 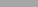: sedentary control animals; 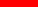: Nogo-A levels in exercised animals; 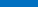: CamKII levels in exercised animals. For comparability of various experimental conditions, the mean of all values of sedentary controls was normalized to 1 and values derived from exercised animals were expressed as percentage of that normalized ROD ± SEM. **(h)** Nogo-A and CamKII levels plotted as a function of running distance during 7 days of exercise, correlated with Nogo-A (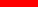) or CamKII (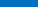) protein levels, respectively, in M1 L2/3 of individual animals. Note that the decrease of Nogo-A and the increase of CamKII levels over time are proportional to the amount of wheel running.

A total of 28 rats was used for the running wheel experiment, with 4 conditions involving 7 rats each. For one rat, only Nogo-A but not CaMKII could be measured, leading to a discrepancy of n = 7 for Nogo-A vs. n = 6 for CaMKII in Fig 1b.

For the skilled forelimb-reaching task (described in section “Motor learning”), 29 additional animals were used.

After a period of handling and habituation to the running wheel, or after antibody pump implantation to recover for the motor learning experiment, respectively, the animals were 7–8 weeks old at the beginning of the experiments (“day 0”). Details on allocation of rats to experimental groups, as well as experimental procedures, are described in more detail under the respective section as follows.

### Running wheel exercise experiment

All rats were single-housed in standard rodent cages. One group of rats (n = 14, non-exercised or “sedentary”) did not receive access to a running wheel and served as control groups for 7 (n = 7), or 14 days (n = 7), respectively. A second group of rats (n = 14) had voluntary access to a running wheel for 7 (n = 7) or 14 days (n = 7), respectively (“exercised” rats). Following the respective time period all animals were perfused for immunohistochemistry as described below. Physical exercising activity was measured as “running distance” (in meters, transformed into kilometers [km] in Fig 1h for better legibility), calculated from the wheel turning frequency (custom made program with LabView software (National Instruments, Austin, TX; RRID:SCR_014325), which recorded each single turn of the running wheel). As no signs of inability to perform physical activity were observed, no animal was excluded from the experiments.

### Immunohistochemistry

For immunohistochemistry, deeply anesthetized animals (Nembutal, 40 mg/kg body weight, intraperitoneally (i.p.); Abbott Laboratories; RRID: SCR_010477) were transcardially perfused with phosphate-buffered saline (PBS; pH 7.4, room temperature) followed by ice-cold fixative (4% paraformaldehyde and 15% saturated picric acid in 0.15 M phosphate buffer (PB, pH 7.4). Brains were removed immediately after perfusion, post-fixed in the same fixative for 12 h at 4°C, and immersed in 10%, 20%, and then 30% sucrose diluted in PBS for cryoprotection before freezing. Coronal slices spanning the motor cortex layers 2/3 (M1 L2/3) and layer 5 (M1 L5) controlling forelimb movements, at 1–2 mm anterior of the bregma [30] were prepared as described previously [31, 32]. Sections (40 μm thick) were cut with a cryostate microtome (model MGW Lauda 1720, Leitz, Wetzlar, Germany) and collected in PBS. Sections were then transferred into custom-made antifreeze solution (15% sucrose and 30% ethylene glycol in 50 mM PB, pH 7.4) and stored at 20°C until used. Free-floating sections were coded and batch processed under identical conditions to minimize staining variability. Sections were incubated overnight at 4°C with primary antibodies against NeuN (1:500; Merck Milipore, Darmstadt, Germany to label neurons and DAPI to label cell nuclei [33], and Nogo-A (“Laura”, 1:250; validated as described before [26]) or CamKII (1:1.000; ThermoFisher Scientific, Upstate, Darmstadt, Germany [34]), respectively, diluted in PBS containing 4% normal goat serum and 0.05% Triton X-100, as described before [26]. After three washes in PBS, the subsequent secondary antibodies were incubated for 2 hours at room temperature in PBS: Alexa Fluor 488 goat anti‐mouse/rabbit (1:200 dilution; Molecular Probes, Göttingen, Germany [35]) and Alexa Fluor 568 goat anti‐mouse/rabbit (1:200; Molecular Probes, Göttingen, Germany; [35]). After rinsing with PBS, the sections were mounted with fluorescence-mounting medium containing DAPI (Vectashield Mounting Medium with DAPI, Vector Laboratories, Burlingame, USA) and stored until subsequent inspection.

### Densitometry

Microscopic analyses of immunohistochemical images were carried out in a blinded manner by a single person who was not involved in any of the experiment and thus unaware of the sample’s group assignment (sedentary control vs. exercise). Images were acquired with a CCD camera (CoolSnap HQ, Photometrics, Tuscon, USA) attached to a Zeiss Axiophot microscope (Zeiss, Oberkochen, Germany), and collected using computer-assisted image analysis software (MCID, Elite Software version 7.0, Imaging Research). For each animal, immunoreactivity (IR) in a given region was obtained from bilateral densitometric measurements, average over 2 sections from the same animal and quantified, taking into account any variability in background staining as described earlier [35], and further evaluated using the software ImageJ (NIH, Bethesda, MD; RRID: SCR_003070). IR was expressed as mean relative optical density (ROD) of all DAPI-labeled cells averaged per brain region (M1 L2/3 and L5) for each animal.

Since RODs for layer M1 L5 did not differ between control and exercised animals, Fig 1g and 1h show only results for M1 L2/3. In order to allow comparison of the markers and time points, the mean ROD of the sedentary control animals was set to 1.0 for each marker (Nogo-A and CaMKII), and the individual values of all animals were converted to percent (%) thereof, shown as mean ± SEM in Fig 1g. Normalized RODs for both Nogo-A and CaMKII were correlated with running distance of individual animals after 7 days of training (Fig 1h).

### Anti-Nogo-A antibody treatment

An anti-Nogo-A (AN)-specific blocking antibody, raised against an 18-aa peptide in the most active region of Nogo-A (Nogo 11c7; RRID: AB_10000211, a kind gift from Novartis Pharma AG, Basel, Switzerland) and a control antibody (mouse IgG Control, AbD Serotec, now Bio-Rad Antibodies; RRID: SCR_008898, Oxford, UK) were used at a concentration of 4.2 mg/ml each and delivered intrathecally as previously described [26]. In brief, after partial laminectomy at the T3 level, a fine intrathecal catheter (32 Ga; Recathco, Alison Park, PA, USA) was inserted into the subarachnoid space from the lumbar level L2/L3 and pushed rostrally to the spinal segment C7, delivering the antibodies continuously from an osmotic minipump (10 μ/h, 2ML1; Alzet Osmotic Pumps) into the cerebrospinal fluid (n = 15 for AN). Animals were treated with the AN antibody for the first 6 days of motor learning and were subjected to the learning task the subsequent 6 days without AN antibody delivery (AN_6_, n = 8), or else received the AN antibody for the entire learning period of 12 days (AN_12_, n = 7). The AN_6_ and AN_12_ groups were designed to allow for discrimination of possible early and late effects of blocking Nogo-A during motor learning. The IgG Control rats (n = 6), receiving only mouse IgG as a control antibody, served to detect possible effects on motor learning of the catheter or pump implantation or of the antibody application procedure per se. In addition, sham-operated rats (“Sham”; n = 8) were also trained and recorded for 12 days.

Sufficient penetration of the applied anti-Nogo-A antibody into the tissue of the rat motor cortex was verified with IHC and Western Blots as previously described (for details, see Fig 7 in [26]).

### Motor learning

AN6, AN12, IgG Control and Sham rats were trained on a complex skilled-forelimb reaching task as previously described [26, 36], with daily training success recorded continuously. Briefly, in the forelimb-reaching training, rats had to grasp a sugar pellet that was placed on a vertical post 1.5 cm away from a slit opening in the cage front, retrievable only by precise forelimb movements (behavioral method adapted from [37] and described in detail in [26]). The rat had to reposition itself prior to each trial by returning to the rear end of the cage, turning around and retrieving the next pellet from the slit opening at the front of the cage. Imprecise movements easily displaced the pellet, which was then lost to the animal. A daily session consisted of 150 trials or a maximum time of one hour for each animal, whichever was reached first. The experimenter was blinded to treatment in being unware of treatment (AN, IgG Control or Sham) throughout all phases of the experiment until completion of data analysis.

### Statistical analysis

**Fig 2:**
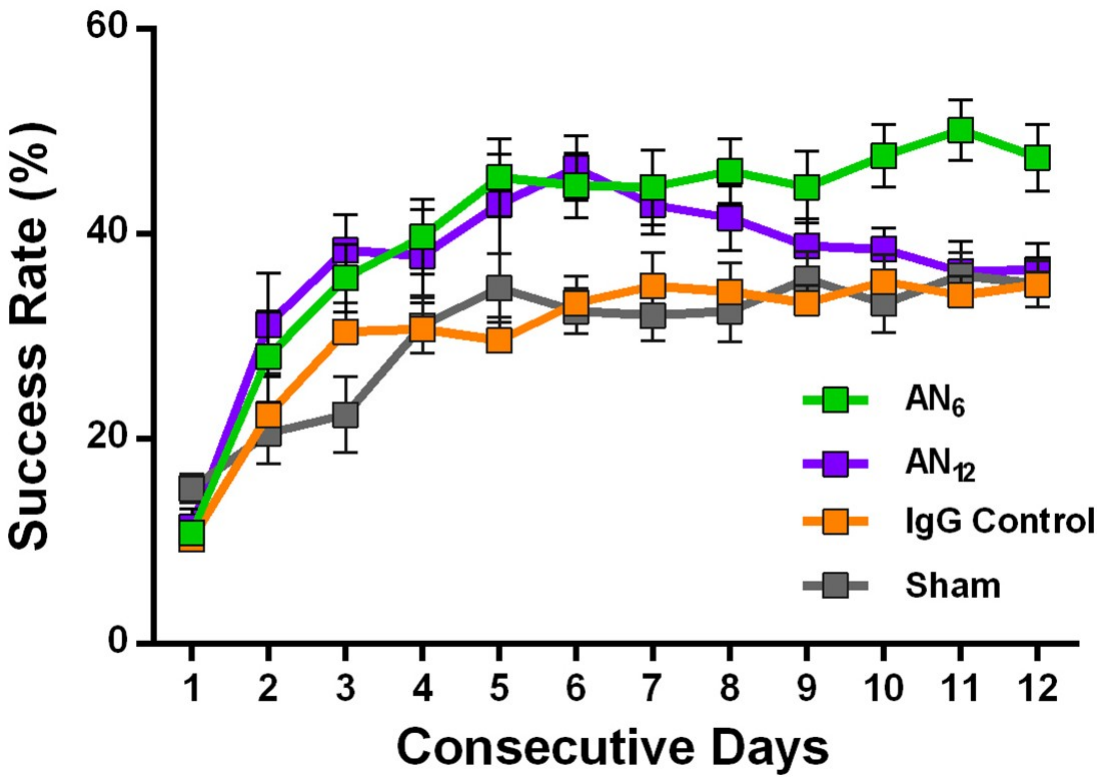
Temporal influence of anti-Nogo-A antibody treatment on *in vivo* motor learning. **(a)** Experimental timeline. **(b)** Rats were exposed to a precision forelimb reaching task while receiving anti-Nogo-A antibodies for the first 6 days (AN_6_: 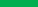; n = 8) of motor learning, or for the entire learning period of 12 days (AN_12_: 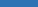; n = 7). Control groups received mouse IgG control antibodies (IgG controls; 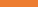; n = 6), or no antibody treatment (Sham: 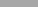; n = 8). Both groups treated with the anti-Nogo-A antibody show significantly higher success rates during the first six days compared to control animals (AN_6_ vs. IgG Control: ***: *P* = 0.02; AN_12_ vs. IgG Control: ***: *P* = 0.01). Note, that in animals, in which treatment with anti-Nogo-A antibodies was terminated after 6 days, a superior success rate was maintained until the end of the consecutive 12-day training period (AN_6_ vs. An12, IgG Control and Sham: ***: *P* = 0.0002), while animals receiving anti-Nogo-A antibodies over consecutive 12 days show a decline in the number of successfully grasped pellets during the second week (day 12: AN_6_ vs. AN_12_: ***: *P* = 0.0002).

All results are presented as mean ± standard error of the mean (SEM). An unpaired Student’s t-test was used for immunohistochemical comparisons shown in Fig 1; since two conditions (assumed to follow normal distribution of underlying samples) were compared at a single time point, no post-hoc correction for false positives was applied. For the motor learning experiments shown in Fig 2, which compared repeated performance of the same animal during 6 or during 12 days, respectively, an 2-way mixed ANOVA followed by Bonferroni’s Multiple Comparison test was carried out in GraphPad Prism 6 software (GraphPad Software, San Diego, CA; RRID: SCR_002798). No test for outliers was performed.

## Results

### Regulation of Nogo-A by running wheel exercise

Semiquantitative densitometric analyses of immunofluorescence images (Relative Optical Density [ROD], see also Table 1) revealed a significant downregulation of Nogo-A after 7 days of voluntary running (Fig 1a, 1e and 1g; [ROD + SEM]: [0.385 ± 0.011] for sedentary controls vs. [0.298 ± 0.011] for exercised animals; *P* = 0.001; n = 7) in M1 L2/3, a layer which is known as the substrate for neuronal plasticity within the motor cortex [32, 38], but not in M1 L5, which does not contribute to plasticity to the same degree as layer 2/3 [38] (Fig 1a, 1e and 1g; [ROD + SEM]: [0.385 ± 0.015] for sedentary controls vs. [0.359 ± 0.015] for exercised animals; *P* = 0.72; n = 7).

**Table 1:** Relative Optical Density [ROD] from Nogo-A and CaMKII protein levels in the motor cortex after 7 respectively 14 days of running wheel exercise.

Values shown in Fig 1a–1h	Sedentary control	Exercised animals	Significance level
	Individual values	Individual values	Student’s t-test
	[mean ± SEM] {normalized mean ± SEM} (n = number of rats)	[mean ± SEM] {normalized mean ± SEM, relative to control} (n = number of rats)	*P-*value
Nogo-A	0.3538	0.3586	
7 days running, M1 L2/3, Fig 1a, 1e and 1g	0.3932	0.2911	*P* = 0.001
	0.3586	0.2869	
	0.3840	0.2785	
	0.3586	0.3038	
	0.4176	0.2700	
	0.4261	0.2953	
	[0.385 ± 0.011]	[0.298 ± 0.011]	
	{1 ± 0.03}	{0.77 ± 0.03}	
	(n = 7)	(n = 7)	
CamKII	0.1739	0.2313	
7 days running, M1 L2/3, Fig 1b, 1f and 1g	0.2189	0.2251	*P* = 0.007
	0.1863	0.2282	
	0.1910	0.2282	
	0.1910	0.2158	
	0.1925	0.2655	
	[0.192 ± 0.006]	[0.232 ± 0.007]	
	{1 ± 0.03}	{1.21 ± 0.04}	
	(n = 6)	(n = 6)	
Nogo-A	0.3434	0.2874	
14 days running, M1 L2/3, Fig 1c and 1g	0.3295	0.3225	*P* = 0.25
	0.2664	0,3014	
	0.2909	0.2839	
	0.2944	0.2909	
	0.3365	0.2523	
	0.3260	0.3260	
	[0.312 ± 0.011]	[0.295 ± 0.009]	
	{1 ± 0.03}	{0.94 ± 0.03}	
	(n = 7)	(n = 7)	
CamKII	0.1400	0.1495	
14 days running, M1 L2/3, Fig 1d and 1g	*P* = 0.41
		0.1522	0.1549
		0.1386	0.1740
		0.1277	0.1399
		0.1645	0.1712
		0.1590	0.1363
		[0.147 ± 0.006]	[0.154 ± 0.006]
		{1 ± 0.04}	{1.03 ± 0.05}
		(n = 6)	(n = 6)
Nogo-A	0.3690	0.4255	
7 days running, M1 L5, Fig 1a, 1c, 1e and 1g	0.3173	0.3597	*P* = 0.72
	0.3726	0.3451	
	0.4076	0.3619	
	0.4092	0.3192	
	0.4204	0.3132	
	0.3959	0.3857	
	[0.385 ± 0.015]	[0.359 ± 0.015]	
	{1 ± 0.03}	{0.93 ± 0.04}	
	(n = 7)	(n = 7)	
CamKII	0.1874	0.2074	
7 days running, M1 L5, Fig 1b, 1d, 1f and 1g	0.1949	0.1807	*P* = 0.65
	0.2138	0.1849	
	0.2101	0.2132	
	0.1521	0.1828	
	0.1952	0.2161	
	[0.192 ± 0.009]	[0.198 ± 0.007]	
	{1 ± 0.05}	{1.03 ± 0.04}	
	(n = 6)	(n = 6)	
Nogo-A	0.3526	0.2789	
14 days running, M1 L5, Fig 1c and 1g	0.2976	0.3231	*P* = 0.72
	0.2834	0.2789	
	0.3379	0.3452	
	0.3038	0.3055	
	0.2596	0.3397	
	0.3397	0.2568	
	[0.311 ± 0.013]	[0.304 ± 0.013]	
	{1 ± 0.04}	{0.99 ± 0.02}	
	(n = 7)	(n = 7)	
CamKII	0.1572	0.1632	
14 days running, M1 L5, Fig 1d and 1g	0.1652	0.1692	*P* = 0.14
	0.1231	0.1412	
	0.1412	0.1792	
	0.1502	0.1422	
	0.1452	0.1724	
	[0.147± 0.006]	[0.161± 0.007]	
	{1 ± 0.02}	{1.09 ± 0.04}	
	(n = 6)	(n = 6)	

Below the individual values and their mean ± standard error of the mean (SEM), also normalized values (mean ± SEM) are provided.

Conversely, the well described neuronal activity marker CamKII [39] was significantly upregulated after 7 days of exercise in M1 L2/3 (Fig 1b, 1f and 1g; [ROD ± SEM]: [0.192 ± 0.006] for sedentary controls vs. [0.232 ± 0.007] for exercised animals; *P* = 0.007; n = 6) but not in ML 5 (Fig 1b, 1f and 1g; [ROD ± SEM]: [0.192 ± 0.009] for sedentary controls vs. [0.198 ± 0.007] for exercised animals; *P* = 0.65; n = 6).

No change in Nogo-A or CamKII protein abundance, respectively, was observed after 14 days of running in M1 L2/3 (Fig 1c, 1d and 1g; Nogo-A: [ROD ± SEM]: [0.312 ± 0.011] for sedentary controls vs. [0.295 ± 0.009] for exercised animals; *P* = 0.25, n = 7; CamKII: [ROD + SEM]: [0.147 ± 0.006] for sedentary controls vs. [0.154 ± 0.006] for exercised animals; *P* = 0.41; n = 6).

Likewise, in cortex layer 5, both Nogo-A and CamKII levels remained unchanged upon 14 days of exercise (Nogo-A: Fig 1c and 1g; [ROD ± SEM]: [0.311 ± 0.013] for sedentary controls vs. [0.304 ± 0.013] for exercised animals; *P* = 0.72; n = 7; CamKII: Fig 1d and 1g; [ROD ± SEM]: [0.147 ± 0.006] for sedentary controls vs. [0.161 ± 0.007] for exercised animals; *P* = 0.14; n = 6).

For better comparability between values shown in Fig 1a and 1c, and between values shown in Fig 1b and 1d, respectively, the mean + SEM of sedentary controls was set to 1.0 and all values of exercised animals were converted to percentages (%) of the respective sedentary control groups in Fig 1g (see also Table 1). Notably, the observed decrease in Nogo-A protein levels as well as the increase in CaMKII protein levels in layer 2/3 of the motor cortex were proportional to the running distance of individual animals after 7 days (Fig 1h).

### Time-dependent influence of Nogo-A on *in-vivo* motor learning

Anti-Nogo-A antibody treatment of rats over 6 consecutive days resulted in higher success rates in the learning task compared to control animals (Fig 2; success rates at day 6: AN_6_: 44.5 ± 4.9%; AN_12_: 42.8 ± 3.7%; IgG Control: 34.9 ± 3.3%; Sham: 32 ± 2.5%; AN_6_ vs. control IgG and vs. sham: *P* = 0.02; AN_12_ vs. IgG Control and vs. Sham: *P* = 0.01; [2-way mixed ANOVA]). No significant difference was observed between the IgG Control and the Sham group (*P* = 0.99; 2-way mixed ANOVA with Bonferroni Multiple Comparison test). During the second half of the testing period, a decline was observed in the success rate of the animals that received continuous anti-Nogo-A antibody treatment over 12 days (AN_12_).

By contrast, in animals in which the application of the anti-Nogo-A antibody was terminated after 6 consecutive days, the elevated success scores acquired during the first 6 days were maintained (Fig 2, success rate at day 12: AN_6_: 47.4 ± 3%, AN_12_: 36.3 ± 3%, Control IgG: 34 ± 1.9%, Sham: 36 ± 2.2%; AN_6_ vs. all other groups: *P* = 0.0002 [2-way mixed ANOVA]. No difference was observed between the AN_12_ group and either control IgG-injected rats or sham-operated animals.

## Discussion

Earlier studies suggested different roles for Nogo-A in learning, as downregulation of Nogo-A signaling came along with improved learning [14, 26], whereas upregulation of Nogo-A signaling was needed to consolidate memories [27, 28]. We now present experimental evidence by exploiting the activity-dependent modulation of Nogo-A in rat motor cortex, that may resolve the apparent discrepancy and unites the conflicting observations into a coherent hypothesis: We found that intense exercise indeed downregulated Nogo-A levels in rat motor cortex, whereas it upregulation at a later stage may enhance memory consolidation. Together, this activity-dependent, bimodal and time-dependent dynamic changes of Nogo-A expression in motor cortex layers 2/3 is likely to bear a functional significance that relates to learning success *in vivo*.

Specifically, we hypothesize that in rat motor cortex layers 2/3, Nogo-A underwent a highly significant downregulation during the first week of running wheel exercise when synaptic plasticity-inhibiting properties of Nogo-A would counteract structural re-arrangements needed to establish new synaptic connections. Such an activity-induced downregulation of Nogo-A levels was not observed in motor cortex layer 5, which is known to not contribute to plasticity to the same degree as does layer 2/3 [38]. By contrast, during the second week of training Nogo-A levels returned to the baseline when the function of Nogo-A as a synaptic plasticity-inhibiting protein seemingly becomes obsolete as memories are consolidated at that time, which is also reflected in the return-to-baseline performance of rats in the forelimb-reaching task.

We verified our observations by pharmacologically silencing Nogo-A function during either the first week or the first two weeks of learning, respectively: Blocking Nogo-A during the first week indeed fostered motor learning. Suppression of Nogo-A in the second week, however, interfered with the learned performance, probably by preventing consolidation of the newly acquired motor skill. The underlying mechanism likely involves strengthening of neuronal plasticity, as has been hypothesized before [14, 26]. Notably, the trial success rate remained elevated during the second week of training, despite the preceding termination of anti-Nogo-A antibody application. This adds an intriguing facet to the potential clinical use of Nogo-A antibodies, namely the possibility that exercise (and thus, motor skill acquisition) induces an overarching effect towards swifter achievement of neuronal homeostasis. Our observations also reveal a presumably critical time window during the first days of motor learning (expressed in the success rate in a forelimb-reaching task), putatively by facilitating structural modifications in neuronal circuity, thus accelerating learning.

Application of Nogo-A antibodies with the aim to improve novel motor skill acquisition would be time-sensitive because the role of exercise-induced modulation of Nogo-A in motor cortex layer 2/3 appears to reverse during the second week of training. The now induced upregulation of this plasticity-inhibiting protein seems to serve as a memory consolidator, shielding newly established circuity from disturbing neuronal noise input. Remarkably, the success rate of rats returned to baseline levels similar to those observed in control animals, in spite of the continued application of Nogo-A antibodies during these two weeks of exercise. Again, this confirms a critical, very early time window—spanning only the first days of novel motor task learning—for successful interference with Nogo-A signaling.

The concomitant anti-correlation between running distance and Nogo-A protein abundance in rat motor cortex 2/3 underlines a functional relationship between physical exercise and Nogo-A protein expression. This backs up behavioral observations, extrapolated from a molecular level, that exercise may principally counteract the limitations of a hard-wired, aged brain.

During development, maturation of neuronal structures and circuits are refined in an experience-dependent manner. In the adult animal, mature CNS networks are much more stable, optimized towards optimal information flow and function [40]. Expression of Nogo-A is highly region-specific and significantly altered from the developing stage to adulthood, especially in areas of the CNS with high plasticity [41, 42], with the notable inclusion of the motor cortex layer 2/3 [32, 38]. Further evidence for a stabilizing function of the Nogo signaling cascade is provided by findings that network connectivity failed to stabilize in Nogo receptor-deficient mice, resulting in expansion of the critical period into adulthood [16, 43]. It is an intriguing speculation that the Nogo expression pattern from development into adulthood might be repeated in learning and consolidation processes: Nogo signaling might act as a fine-tuned gatekeeper in the CNS, with Nogo-A being temporarily downregulated during learning to permit structural rearrangements (“opening the gate”), and at later stages being upregulated to consolidate the newly established circuitry (“closing the gate”). We therefore feel that we can experimentally support the hypothesis that Nogo-A acts as a dynamic plasticity gatekeeper in memory formation and consolidation homeostasis.

Optimally tuned supplementation of neuronal plasticity inhibitors opens powerful avenues to re-establish lost functionality after CNS injury. Nogo-A function-blocking antibodies have been applied in spinal cord injury, multiple sclerosis and amyotrophic lateral sclerosis in pilot clinical trials [44–46]. The above-mentioned, bimodal and time-dependent role of Nogo-A in motor learning has, in our view, a high relevance for clinical trials: Whereas treatment with anti-Nogo-A antibodies may accelerate the downregulation of the Nogo-A protein induced by physical activity and might thus boost recovery from CNS injury during a short time window [26], inadequately timed application of the same antibody may adversely influence consolidation of motor skills by down-regulating Nogo-A at a stage where it is required as a synaptic plasticity gatekeeper. Thus, timing the anti-Nogo-A antibody application to the ideal temporal window will be crucial for clinical approaches aimed at neuronal rehabilitation.

## Supporting information

S1 File (DOCX)

## Abbreviations

AN_12_: Anti-Nogo-A applied for 12 days
AN_6_: Anti-Nogo-A applied for 6 days
CamKII: calmodulin kinase II
CNS: central nervous system
IR: immunoreactivity
PBS: phosphate-buffered saline
ROD: Relative Optical Density
RRID: Research Resource Identifier (see https://scicrunch.org/resources)
