# Supplementary material for: Exercise-induced Nogo-A influences rodent motor learning in a time-dependent manner
Source: PLoS One. 2021 May 5;16(5):e0250743. doi: 10.1371/journal.pone.0250743 (PMC8099082; doi:10.1371/journal.pone.0250743)
Supplement: S1 File — (DOCX) [file pone.0250743.s001.docx]

**S1 File to:**

**Exercise-induced Nogo-A influences rodent motor learning in a time-dependent manner**

Jörg Stehle^1,2,*^, Zhiyuan Sheng^1*^, Laura Hausmann^3*^, Phillip Bechstein^2^, Oliver Weinmann^4,5^, Juha Hernesniemi^1^, Joseph S. Neimat^6^, Martin E. Schwab^4,5^, Ajmal Zemmar^1,4,5,a^

^1^ Department of Neurosurgery, People’s Hospital of Zhengzhou University, Henan Provincial People´s Hospital, Henan University People’s Hospital, Henan University School of Medicine, 7 Weiwu Road, Zhengzhou, China 450000

^2^ Dr. Senckenbergische Anatomie, Goethe-University Frankfurt, D-60590 Frankfurt am Main, Germany

^3^ University Hospital RWTH Aachen, Department of Neurology, Pauwelsstrasse 30, 52074 Aachen, Germany

^4^ Brain Research Institute, University of Zurich, 8057 Zurich, Switzerland

^5^ Department of Biology and Department of Health Sciences and Technology, ETH Zurich,

8057 Zurich, Switzerland

^6^ Department of Neurosurgery, University of Louisville, School of Medicine, 200 Abraham Flexner Way, Louisville, KY 40202, USA

* These authors contributed equally to this work

^a^Correspondence should be addressed to: Ajmal Zemmar: ajmal.zemmar@gmail.com

**ORCiD Identifiers (https://orcid.org/):**

Jörg H. Stehle: <https://orcid.org/0000-0003-3300-1379>

Laura Hausmann: <https://orcid.org/0000-0003-0174-7248>

Juha Hernesniemi: <https://orcid.org/0000-0001-8163-8804>

Joseph S. Neimat: https://orcid.org/0000-0002-1114-8938

Ajmal Zemmar: <https://orcid.org/0000-0003-1463-3338>

For data points as presented in Figure 1 a)-d), please refer to **Table 1** in the main manuscript.

**Supplementary Table 1**: Normalized Relative Optical Density (ROD) for exercising days 0, 7 and 14, as presented in Figure 1 g.

| **Sedentary control** | Days of exercise | | |
| --- | --- | --- | --- |
|  | 0 | 7 | 14 |
| Animals | 0.9850249  1.111481  0.905158  0.9983361 | 0.9216301  1.020376  0.9326019  0.9984326  0.9326019  1.086207  1.108150 | 1.095847  1.051118  0.9057508  0.6821086  0.9392971  1.285942  1.039936 |

| **Exercise Nogo-A** | Days of exercise | | |
| --- | --- | --- | --- |
|  | 0 | 7 | 14 |
| Animals | 0.9450915  0.9983361  1.018303  1.291181 | 0.9326019  0.7570533  0.7460815  0.7241379  0.7899687  0.7021943  0.7680251 | 0.7603834  1.218850  1.062300  0.9057508  0.9057508  0.615016  1.151757 |

| **Exercise CamKII** | Days of exercise | | |
| --- | --- | --- | --- |
|  | 0 | 7 | 14 |
| Animals | 0.8403361  1.055462  0.9546219  1.122689 | 1.203230  1.170929  1.187079  1.187079  1.122476  1.380888 | 1.016949  1.053929  1.183359  1.201849  1.164869  0.927319 |

**Supplementary Table 2**: Normalized Relative Optical Density (ROD) for Nogo-A and Calmodulin Kinase II (CaMKII) as presented in Figure 1 h).

|  | | |  |
| --- | --- | --- | --- |
| Running distance (km) | Nogo-A ROD, M1 Layer 2/3, 7d of exercise | CamKII ROD, M1 Layer 2/3,  7d of exercise | |
| 12 | 0.932600 | 1.122400 | |
| 23 | 0.789900 | 1.170900 | |
| 24 | 0.768000 | 1.187000 | |
| 25 | 0.757000 | 1.182100 | |
| 32 | 0.746000 | 1.187000 | |
| 37 | 0.724100 | 1.203200 | |
| 69 | 0.702100 | 1.380900 | |

**Supplementary Table 3**: Success rates for consecutive days (1-12) of forelimb reaching task training for n = 6-8 rats for the various experimental conditions as depicted in Figure 2.

| Mouse IgG Control rats (n = 6) | | | | | | |
| --- | --- | --- | --- | --- | --- | --- |
| Day of exercise | Rat 1 | Rat 2 | Rat 3 | Rat 4 | Rat 5 | Rat 6 |
| 1 | 12.37 | 3.33 | 14.04 | 8.76 | 8.49 | 13.98 |
| 2 | 12.00 | 21.33 | 37.33 | 12.00 | 30.00 | 21.33 |
| 3 | 28.00 | 18.00 | 32.00 | 36.67 | 36.67 | 31.33 |
| 4 | 20.67 | 30.67 | 22.67 | 41.33 | 32.00 | 36.67 |
| 5 | 35.33 | 22.00 | 24.67 | 34.00 | 34.00 | 27.33 |
| 6 | 30.00 | 24.67 | 35.33 | 28.67 | 41.33 | 39.33 |
| 7 | 25.33 | 28.00 | 39.33 | 43.33 | 43.33 | 30.00 |
| 8 | 42.00 | 34.00 | 22.67 | 40.00 | 35.33 | 32.00 |
| 9 | 27.33 | 36.67 | 34.00 | 29.33 | 38.00 | 34.00 |
| 10 | 34.67 | 44.00 | 26.67 | 33.33 | 42.00 | 31.33 |
| 11 | 29.33 | 31.33 | 42.00 | 31.33 | 36.67 | 33.33 |
| 12 | 24.67 | 41.33 | 34.00 | 35.33 | 40.00 | 34.67 |

| Sham Control rats (n = 8) | | | | | | | | |
| --- | --- | --- | --- | --- | --- | --- | --- | --- |
| Day of exercise | Rat 1 | Rat 2 | Rat 3 | Rat 4 | Rat 5 | Rat 6 | Rat 7 | Rat 8 |
| 1 | 17.95 | 15.45 | 15.38 | 12.12 | 14.00 | 11.68 | 23.21 | 11.24 |
| 2 | 19.33 | 31.33 | 22.00 | 12.00 | 26.00 | 30.67 | 12.00 | 10.67 |
| 3 | 36.67 | 20.67 | 14.00 | 10.67 | 14.67 | 35.33 | 15.33 | 31.33 |
| 4 | 30.67 | 36.00 | 22.67 | 44.00 | 38.00 | 23.33 | 24.00 | 29.33 |
| 5 | 32.67 | 18.67 | 34.67 | 43.33 | 28.67 | 50.67 | 34.67 | 34.00 |
| 6 | 20.67 | 34.67 | 32.00 | 41.33 | 31.33 | 34.67 | 27.33 | 37.33 |
| 7 | 34.67 | 27.33 | 31.33 | 45.33 | 23.33 | 36.67 | 24.67 | 32.67 |
| 8 | 28.67 | 35.33 | 38.00 | 21.33 | 44.67 | 30.00 | 22.00 | 39.33 |
| 9 | 32.00 | 43.33 | 34.67 | 42.67 | 31.33 | 37.33 | 20.67 | 42.67 |
| 10 | 26.67 | 40.00 | 33.33 | 25.33 | 28.67 | 47.33 | 25.33 | 38.67 |
| 11 | 35.33 | 26.00 | 42.67 | 38.67 | 41.33 | 27.33 | 35.33 | 40.67 |
| 12 | 32.67 | 35.33 | 34.00 | 41.33 | 30.67 | 46.00 | 25.33 | 35.33 |

| Anti-Nogo-A antibody treatment, 6 days (n = 8) | | | | | | | | |
| --- | --- | --- | --- | --- | --- | --- | --- | --- |
| Day of exercise | Rat 1 | Rat 2 | Rat 3 | Rat 4 | Rat 5 | Rat 6 | Rat 7 | Rat 8 |
| 1 | 6.74 | 8.70 | 9.92 | 8.66 | 10.59 | 17.65 | 12.96 | 11.33 |
| 2 | 24.67 | 18.00 | 31.33 | 44.67 | 26.00 | 14.00 | 17.33 | 48.00 |
| 3 | 42.67 | 32.67 | 34.67 | 28.67 | 40.67 | 18.00 | 40.67 | 47.33 |
| 4 | 46.67 | 34.00 | 45.33 | 30.00 | 49.33 | 22.00 | 51.33 | 38.67 |
| 5 | 41.33 | 48.67 | 59.33 | 28.00 | 45.33 | 37.33 | 60.67 | 43.33 |
| 6 | 53.33 | 43.33 | 52.67 | 38.67 | 46.00 | 28.00 | 54.00 | 41.33 |
| 7 | 42.67 | 31.33 | 54.00 | 46.67 | 57.33 | 27.33 | 50.00 | 46.67 |
| 8 | 31.33 | 40.67 | 54.67 | 43.33 | 59.33 | 52.00 | 45.33 | 42.00 |
| 9 | 38.67 | 52.00 | 28.67 | 48.00 | 55.33 | 42.67 | 56.00 | 34.67 |
| 10 | 48.00 | 43.33 | 39.33 | 61.33 | 54.67 | 34.67 | 46.67 | 52.67 |
| 11 | 55.33 | 45.33 | 38.00 | 58.67 | 62.00 | 50.67 | 41.33 | 49.33 |
| 12 | 32.67 | 42.00 | 51.33 | 41.33 | 57.33 | 48.00 | 45.33 | 61.33 |

| Anti-Nogo-A antibody treatment, 12 days (n = 7) | | | | | | | |
| --- | --- | --- | --- | --- | --- | --- | --- |
| Day of exercise | Rat 1 | Rat 2 | Rat 3 | Rat 4 | Rat 5 | Rat 6 | Rat 7 |
| 1 | 12.62 | 8.33 | 6.35 | 14.17 | 18.00 | 5.04 | 14.96 |
| 2 | 18.67 | 50.67 | 16.00 | 38.00 | 22.00 | 44.67 | 28.00 |
| 3 | 42.00 | 49.33 | 38.00 | 45.33 | 25.33 | 26.67 | 42.00 |
| 4 | 34.00 | 38.67 | 27.33 | 56.00 | 30.67 | 52.67 | 25.33 |
| 5 | 48.00 | 46.00 | 22.00 | 54.67 | 27.33 | 49.33 | 52.67 |
| 6 | 58.67 | 33.33 | 40.67 | 48.67 | 53.33 | 42.67 | 47.33 |
| 7 | 49.33 | 45.33 | 32.67 | 52.00 | 46.00 | 34.00 | 40.00 |
| 8 | 56.67 | 35.33 | 38.67 | 40.00 | 49.33 | 36.67 | 34.00 |
| 9 | 50.00 | 36.67 | 43.33 | 26.67 | 40.67 | 35.33 | 38.67 |
| 10 | 42.00 | 45.33 | 39.33 | 35.33 | 37.33 | 41.33 | 28.67 |
| 11 | 47.33 | 43.33 | 36.00 | 28.00 | 40.67 | 31.33 | 27.33 |
| 12 | 45.33 | 37.33 | 30.67 | 30.67 | 42.67 | 40.67 | 28.00 |
